# Feasibility of using a transmission ion chamber for QA tests of medical linear accelerators

**DOI:** 10.1002/acm2.14245

**Published:** 2024-01-09

**Authors:** Makan Farrokhkish, Andrew J. Veres, Luis E. Fong de los Santos, John J. DeMarco, Mohammad K. Islam

**Affiliations:** ^1^ Department of Medical Physics Princess Margaret Cancer Centre Toronto Ontario Canada; ^2^ Department of Radiation Oncology Mayo Clinic Rochester Minnesota USA; ^3^ Department of Radiation Oncology Cedars‐Sinai Medical Center Los Angeles California USA; ^4^ Department of Radiation Oncology University of Toronto Toronto Ontario Canada

**Keywords:** IQM, Linac QA, transmission chamber

## Abstract

**Purpose:**

To study the feasibility of using the Integral Quality Monitoring (IQM) system for routine quality assurance (QA) of photon beams.

**Methods:**

The IQM system is a commercially available dose delivery verification tool, which consists of a spatially sensitive large area transmission ion chamber, mounted on the Linac collimator, and a calculation algorithm to predict the signals in response to radiation beams. By comparing the measured and predicted signals the system verifies the accuracy of beam delivery. The ion chamber unit is a battery powered system including a dual‐electrometer, temperature and pressure sensors, and inclinometers. The feasibility of using the IQM system for routine QA tests was investigated by measuring constancy values of beam parameters, with specially designed tests fields, and comparing them with those determined by a conventional system.

**Results:**

The sensitivity of the beam output constancy measurements by the IQM system was found to agree with those measured by a Farmer type ion chamber placed in water phantoms to within 0.1% for typical daily output variation of ± 0.5% and ± 1%. The beam symmetry was measured with a 4 cm × 4 cm aperture at multiple off‐axis distances and was found to have a highly linear relationship with those measured in a water phantom scan for intentionally introduced asymmetry between ‐3% and +3%. The beam flatness was measured with a two‐field ratio method and was found to be linearly correlated with those measured by water phantom scan. The dosimetric equivalent of a picket fence test performed by the IQM system can serve as a constancy check of the multileaf collimator (MLC) bank positioning test.

**Conclusions:**

The IQM system has been investigated for constancy measurements of various beam parameters for photon beams. The results suggest that the system can be used for most of the routine QA tests effectively and efficiently.

## 1 INTRODUCTION

The safety and accuracy of high precision treatments such as intensity modulated radiation therapy (IMRT) and volumetric modulated arc therapy (VMAT) techniques rely on the tight performance tolerances of the medical linear accelerator (Linac). The International Commission on Radiation Units and Measurements (ICRU) guidelines recommend the delivered dose to the patient to be within ± 5% of the prescribed dose.^1^ To meet this standard, monitoring the accuracy of Linac performance and fulfillment of quality standards is of high importance. As a result, comprehensive quality assurance (QA) protocols are developed and implemented in each radiotherapy department that follow the quality standards set by community‐of‐practice organizations such as International Atomic Energy Agency (IAEA)^2^ and American Association of Physicists in Medicine (AAPM)^3^, ^4^ task groups. These protocols require daily, monthly, and annual QA checks of various dosimetric and mechanical performance parameters.

The Linac QA measurements are performed using a variety of available quality control (QC) devices such as Farmer type ion chambers, 2‐D detector arrays, electronic portal imaging devices, and water phantoms (WP). The selection of the QC tools depends upon their availability, cost, suitability, efficiency, sensitivity, and versatility. In this study, we describe the potential utilization of a recently introduced real‐time patient‐specific treatment QC tool called the Integral Quality Monitoring (IQM) system for routine QA of Linac parameters.

The IQM system (manufactured by iRT, Germany), described previously by Islam et al.,^5^ consists of a spatially sensitive large‐area transmission chamber mounted on the Linac collimator, and a calculation algorithm to predict the response signal to a radiation field. By comparing the predicted and measured “dose‐area‐product” signal, the system can validate the accuracy of static and dynamic dose delivery for each beam segment in real‐time. The IQM chamber assembly has a built‐in rechargeable battery, electrometer electronics for dosimetry, temperature and pressure sensor for environmental correction, and an inclinometer to independently measure gantry and collimator angle.

The IQM system is primarily developed to validate the accuracy of beam delivery both as a real‐time monitor (i.e., during treatment), as well as for pre‐treatment validation of patient specific treatment plans. The characteristics and performance of IQM system for IMRT and VMAT treatment patient‐specific QA has been investigated and compared to other devices.^6^, ^7^, ^8^, ^9^, ^10^, ^11^, ^12^, ^13^ Since the IQM detector is robustly mounted at the Linac collimator, and works with well‐understood ionization chamber principles, it is anticipated that the system's large transmission chamber may potentially be utilized as an efficient Linac QA device for a selected set of dosimetric parameters.

To investigate the suitability of using the IQM system for machine QA, a collaborative workgroup consisting of three clinical centers has been formed. It is recognized that the IQM system provides only a single signal of a “dose‐area‐product” for each beam segment measurement, as opposed to multiple point measurements provided by conventional QC tools, such as a 2‐D detector array. We hypothesize that the IQM signals of a specially designed field aperture, or a combination of apertures can be utilized to provide the constancy measures of a selected set of QC parameters. For example, a central axis point dose measured by a conventional dosimetry system for output constancy can be easily correlated by an IQM signal measured for a square field; and an off‐axis point dose measured by a point dosimetry system (e.g., 2‐D array detector) can be correlated with an IQM signal measured for an off‐axis small square field aperture. Considering this, the machine QA tests investigated here included photon output constancy, output constancy versus gantry angle, output versus nominal dose‐rate, beam symmetry and flatness, and multileaf collimator (MLC) positioning accuracy. In this work each center used their own IQM system to perform measurements of some of the QC tests on their respective linear accelerators. However, the beam symmetry and flatness investigation were made in only one center, where expertise and opportunities were available to make intentional changes to beam symmetry and flatness for the purpose of the experiment.

Performance and sensitivity of the IQM system were assessed by examining its response to various dosimetric errors that were either inherent to the Linac, or errors that were deliberately introduced. The response of the IQM system was then compared to a set of reference measurements taken with conventional equipment.

## 2 METHODS AND MATERIALS

**FIGURE 1:**
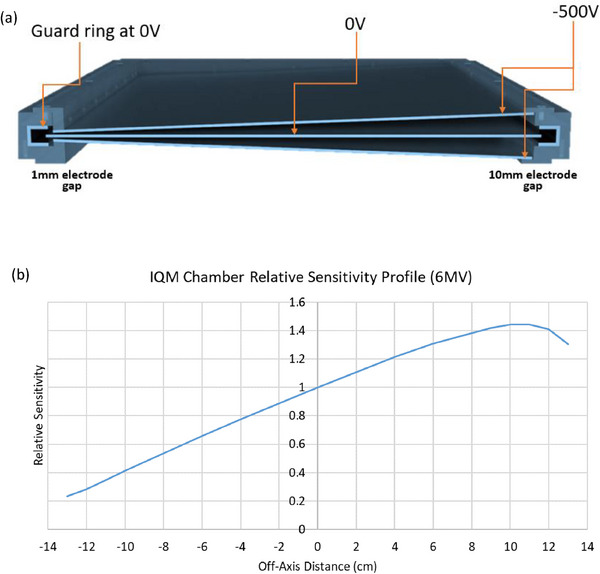
Cross‐section of the Integral Quality Monitor (IQM) transmission chamber along the transverse axis: (a) The inclination of the polarizing electrodes with respect to the collector varies the plate separation from 1 to 10 mm, creating an intrinsic spatial sensitivity over the entire length of the chamber. (b) The spatial sensitivity profile for a 6 MV beam.

The IQM system consists of a single large‐area (26.5 × 26.5 cm^2^) transmission ion chamber that encompasses the entire radiation beam area when mounted on the Linac collimator. The transmission chamber consists of a collector plate positioned between two polarizing plates, each 1 mm in thickness. The polarizing plates are inclined with respect to the collector, varying the plate separation from 1 to 10 mm across the length of the chamber to create a spatial sensitivity gradient in the direction of the MLC motion illustrated in Figure 1. An internal power supply applies a fixed bias of −500 V to the collector electrode.

The chamber housing incorporates a dual channel electrometer to measure the radiation signal and a tri‐axial Micro‐Electro‐Mechanical (MEMs) accelerometer for independent verification of Linac gantry and collimator angles. The accelerometer‐electrometer data pair is reported via Bluetooth communication at a rate of 5 Hz to a transceiver located inside treatment room. The transceiver relays the data to a software, which displays and compares each measured segment against reference data throughout the beam delivery. The entire system is powered by a set of rechargeable lithium Ion batteries.

IQM measurements were accompanied by a set of independent reference measurements that were either performed simultaneously or shortly thereafter depending on the type of test. The reference measurements were typically done using NE‐2571 and FC65‐G chambers placed inside either a solid water phantom or with a water phantom (WP) system. All IQM and reference measurements were repeated for a minimum of three times, and percent standard deviation (%STDEV) was calculated to assess measurement reproducibility.

Measurements of all the selected set of QA tests except beam flatness were performed on Varian TrueBeam (Varian Medical Systems, Palo Alto, CA) Linac equipped with 120 Millennium MLC leaves, with 6 MV, 10 MV, 18 MV, 6 FFF, and 10 FFF beam energies. The beam flatness test was performed on a recently decommissioned Elekta Agility (Elekta AB, Stockholm, Sweden) unit, as it allowed unrestricted beam adjustments for the purpose of the experiments.

### 2.1 Beam output

The sensitivity of the IQM system to output variation by ± 1% was previously investigated for 6 MV beams.^13^ However, in this work, sensitivity to typical variations of daily output was expanded for all beam energies, and compared to reference measurements. Simultaneous measurements were performed with the IQM system and a reference ion chamber (placed at isocenter and at the depth of *d*_max_ in a 30 × 30 cm^2^ solid water phantom) for a field setting of 20 × 20 cm^2^ and 100 MU. This field size is chosen instead of a conventional 10 × 10 cm^2^ field to reduce the effect of beam collimator positioning error on the dose‐area‐product signal. The beam output variation was simulated by varying MU settings by ± 0.5% and ± 1.0%. Subsequently, the changes in signals for the IQM chamber and the reference chamber due to the variations in the beam output were analyzed and compared.

### 2.2 Beam output versus gantry angle

Since the IQM detector is easily mounted at the Linac collimator with high setup reproducibility, and rotates with gantry, the feasibility of beam output measurement at various gantry angles has been investigated for all beam energies. Similar to the previous section, measurements were performed by the IQM system and a reference ion chamber placed inside of a cylindrical solid water phantom at a depth of 10 cm. The phantom was rested on a Varian head extension couch S‐frame making sure that the center of the ion chamber coincides with the iso‐center. The head extension does cause a negligible beam attenuation for measurements at gantry 180°. The signals for a 100 MU beam and field size of 20 × 20 cm^2^ was measured by both detectors simultaneously at each cardinal gantry angle. Signals were normalized to the corresponding reading at gantry angle zero and compared.

### 2.3 Beam output versus dose rate

Photon output constancy with nominal dose rate is checked annually to check the Linac dose delivery control system and to ensure that the definition of 1 MU is near constant at various dose rates. The dose‐rate dependency of the IQM system was previously investigated for a 6 MV beam and minimal dependence was observed by Hoffman et al.^13^ In this study, IQM and reference ion chamber were used to measure the Linac's inherent output variation with dose‐rate for 6 and 18 MV energies as well as 6 FFF and 10 FFF beams. The signals for a 100 MU beam were acquired with both detectors at various nominal dose rates using a 10 × 10 cm^2^ field size. The measured signals were then normalized to those acquired at maximum dose‐rate for each corresponding detector, and compared.

### 2.4 Beam symmetry

A test field was developed to measure beam symmetry using the IQM system. The test field consists of jaw‐defined 4 × 4 cm^2^ field segments located at 6 and 12 cm away from the beam center. This size of the field was chosen to represent a “point dosimetry”; a smaller field size would have larger uncertainties in signal due to position errors of the jaws, while a larger field size would cause a larger signal averaging effect. By delivering the off‐axis beams and measuring the signals at all cardinal collimator angles, IQM transmission chamber samples the radiation beam in the radial and transverse axes.

**FIGURE 2:**
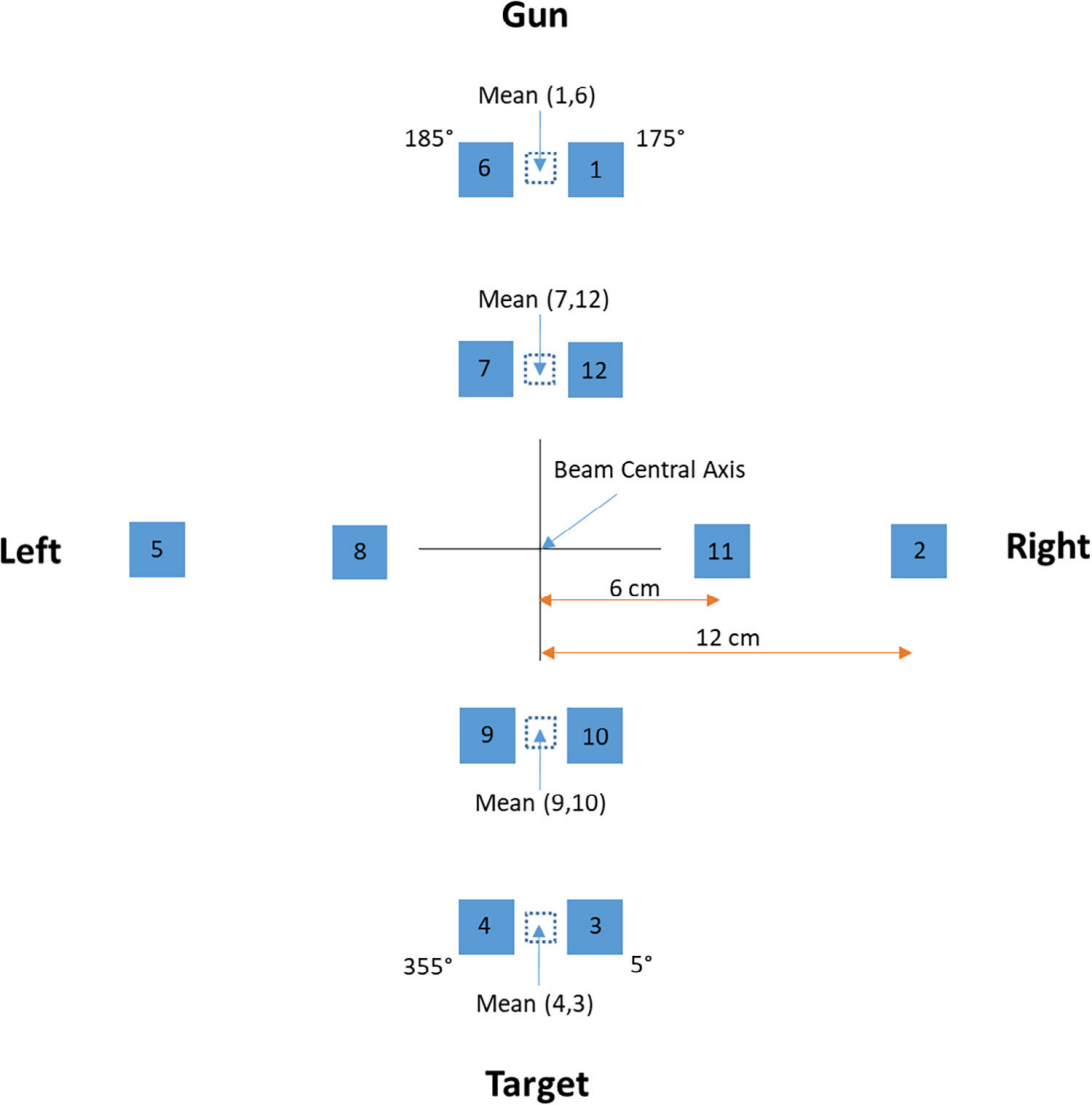
Illustration of the composited beams‐eye‐view of Integral Quality Monitor (IQM) beam symmetry measurement apertures: 4 × 4 cm^2^ field segments (solid blue box) at 6 and 12 cm off‐axis locations (defined at the isocenter) are delivered at all cardinal collimator angles, sampling the beam along two circular paths with radius 6 and 12 cm. The number inside each box indicates the beam segment delivery sequence. The dashed blue square at collimator 0° and 180° represents the average signal of the neighboring beam openings.

The functionality of the test field and IQM system for beam symmetry verification was evaluated for a non‐clinical 10 MV beam. The beam symmetry was deliberately varied approximately in the range of −3% to +3% (as per IEC‐60976 definition 100% to 103%) with 1% increments by adjusting the radial angle (R) and transverse angle (T) values in the Linac beam control system. The final beam symmetry as reported by the Linac monitoring chamber after each beam adjustment was defined as Set up symmetry ($Set_{SYM}$). The test field was delivered after beam symmetry adjustment. Figure 2 illustrates the beam delivery and corresponding measurement sequence for symmetry measurement with the IQM system. An approach similar to that of the IEC‐60976 protocol was used to calculate the transverse and radial beam symmetry as defined by Equation (1):

(1) $$IQM_{SYM}^{Transverse}=Average\left(IQM_{SYM}^{6\;cm},IQM_{SYM}^{12\;cm}\right)$$

where,

$$IQM_{SYM}^{6\;cm}=\left(\frac{Q_{90}^{6\;cm}}{Q_{270}^{6\;cm}}-1\right)\times 100$$

$$IQM_{SYM}^{12\;cm}=\left(\frac{Q_{90}^{12\;cm}}{Q_{270}^{12\;cm}}-1\right)\times 100$$

The $Q_{c}^{d}$ represents the IQM signal measured with the 4 × 4 cm^2^ aperture at the off‐axis distance of *d* cm, with a collimator angle of *c*. The symmetry calculated by Equation (1) reports both magnitude and direction of beam symmetry. For example, a positive beam symmetry implies a higher dose rate on the right‐hand side of the beam profile. In addition, Equation (1) implies a value of 0% for a perfectly symmetric beam. Similar equations were applied for the calculation of $IQM_{SYM}^{Radial}$. However, since the collimator angle rotation of a Varian TrueBeam Linac does not allow setting of a collimator angle at exactly 180°, the average measurements at collimator angles 175° and 185° were taken to represent the corresponding IQM signals at collimator 180°. For consistency, this was repeated for collimators 5° and 355° to estimate signal at collimator 0°. A positive value in $IQM_{SYM}^{Radial}$ indicates a detection of higher dose rate at the gun‐side of the beam.

Following the measurement of symmetry with the IQM system, immediately afterwards the reference symmetry was measured by a WP system. WP scans were acquired for a 30 × 30 cm^2^ field size using the Blue Phantom and CC13 ion chambers (manufactured by IBA Dosimetry GmbH, Schwarzenbruck, Germany) at the depth of *d*_max_ and at (source to surface distance) SSD of 100 cm. The obtained WP beam profiles were analyzed by the scanner software using the IEC‐60976 protocol as shown by Equation (2):

(2) $$\begin{array}{ccc} Symmetry_{IEC} & = & MAX\left(\left|\frac{D_{Right}}{D_{Left}}\right|,\left|\frac{D_{Left}}{D_{Right}}\right|\right)\\ & & \times \;100\left(Within\;80\%\;of\;Field\;Size\right) \end{array}$$

where Symmetry is the maximum dose ratio measured at any two opposite and equidistant points within 80% of the radiation field. For the purpose of direct comparison of symmetry measured by the IQM system and those measured by WP, Equation (2) was slightly modified and calculated using Equation (2.1) and Equation (2.2):

(2.1) $$\begin{array}{ccc} WP_{SYM}^{Transverse} & = & MAX\left(\frac{D_{Right}}{D_{Left}}-1\right)\\ & & \times \;100\left(Within\;80\%\;of\;Field\;Size\right) \end{array}$$

(2.2)

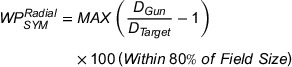

This modification allows assignment of direction to beam symmetry in both positive and negative, and to 0% for a perfectly symmetric beam. The measured $IQM_{SYM}$ for each beam setting was compared to $Set_{SYM}$, and $WP_{SYM}$.

### 2.5 Beam flatness

The typical beam flatness measurement by comparing the maximum and minimum of beam dose profile is not feasible with the IQM system since it utilizes a single large transmission chamber. An alternative method of using ratios of large and small field measurements was investigated as a measure of beam flatness. A field size of 35 × 35 cm^2^ was used to capture both the central area of the field as well as most of the peripheral area containing the beam horns, and a field size of 10 × 10 cm^2^ was used to capture the central area containing the minimum point of the relative dose profile. The flatness constancy measured by the IQM system (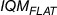) is then defined by Equation (3):

(3) $$IQM_{FLAT}=\frac{Q_{35X35}}{Q_{10X10}}$$

where $Q_{35X35}\;and\;Q_{10X10}$ represent the IQM signals for field sizes of 35 × 35 cm^2^ and 10 × 10 cm^2^ respectively, with a fixed (e.g., 100 MU of) beam delivery. For this section, a recently decommissioned Elekta Infinity (Elekta, Stockholm, Sweden) Linac equipped with Agility 160‐MLC model, and available clinical beam energies of 6 and 18 MV was used. Sensitivity of the IQM system to changes in beam energy, and consequently to the beam flatness, was studied for 6 and 18 MV photon beams, and compared to the beam flatness measured by WP scans.

In this study, beam energy was changed from its baseline by adjusting the bending magnet current, gun current, and magnetron power output. The bending magnets were cycled after each beam parameter change to reduce hysteresis. Beam profiles for a 35 × 35 cm^2^ field size were acquired with WP using the same setup as the previous section after each beam energy change. Beam flatness measured by water phantom 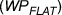 was defined using the IEC‐60976 protocol Equation (4):

(4) $$WP_{FLAT}=\frac{D_{Max}}{D_{Min}}\left(Within\;80\%\;of\;Field\;Size\right)$$

where *D_Max_* and *D_Min_* are the maximum and minimum dose values on the measured beam profile.

After the WP scan, the IQM chamber was attached to the collimator and the signals for 10 × 10 cm^2^ and 35 × 35 cm^2^ field sized were acquired. Measured 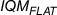 was plotted against $WP_{FLAT}$ to define the relationship between the two measured beam flatness, and a tolerance on IQM beam flatness measurement in line with current radiotherapy standards was defined.

### 2.6 Static MLC positioning accuracy

**FIGURE 3:**
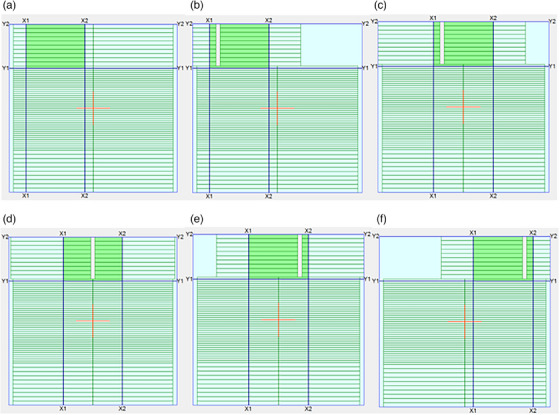
Static MLC positioning accuracy test field for the first row formed by leaf pairs 51 to 60. All MLCs start at position zero (a). The 1 × 10 cm^2^ slit is delivered at 0 (d), ± 5 (c and e), and ± 14 cm off‐axis positions (b and f). The slits expose the IQM system in the gradient direction. The beam delivery sequence is repeated for the remaining three rows. IQM, Integral Quality Monitor; MLC, multileaf collimator.

The MLC positioning accuracy test is conventionally performed by inspection of a Picket‐Fence pattern of narrow slits created by MLC at various sequential positions on film or EPID.^14^, ^15^, ^16^, ^17^ In this work, we investigate a dosimetric equivalent of the Picket‐Fence test utilizing the spatial sensitivity of the IQM chamber along the direction of the MLC motion. This test is performed by dividing the 40 cm MLC bank into four rows of Picket Fences, with a narrow slit being 10 cm in length and 1 cm wide. Since the Linac is equipped with the 120 MLC Millennium collimator model, the narrow slit for first and last rows are formed by the 10 mm wide leaves (leaf pairs 51−60 and 1−10, respectively), while the slit for the second and third rows are formed by the 5 mm wide leaves (leaf pairs 31−50 and 11−30 respectively). The narrow slit is moved to 0, ± 5, and ± 14 cm off‐axis positions within each row with a beam delivery of 25 MUs at each position. Figure 3 shows the beam delivery sequence for the first row. Initially all leaves were in the closed position such that all slits are formed from the same initial MLC position as shown in Figure 3a. This was done to maintain the same MLC positioning error as a function of traveled distance. Each MLC slit was confined inside a 14 × 11 cm^2^ jaw defined field size to keep the background signal due to MLC transmission to near constant for all off axis slits. The X and Y jaws were placed 5 mm behind the MLCs whenever possible to minimize the effect of jaw positioning uncertainties on the overall opening area.

IQM signals are captured as the baseline values for constancy measurements. In addition, the sensitivity of these measurements is determined by introducing systematic (MLC bank) errors in leaf positioning. The tests are also repeated for various gantry angles. Although the MLC positioning test results with the IQM system are not identical to those performed by film and EPID, these tests can be easily and frequently performed for dosimetric constancy of MLC positions, and therefore can be used at least as a supplementary test. An IMRT field was developed using multiple segments to assess the MLC positioning test for Varian Millennium 120 MLC model.

### 2.7 Dynamic MLC positioning accuracy

The accuracy of a dynamic beam delivery depends on the ability of the two opposing MLC banks to continuously maintain the gap defined in the treatment plan throughout the delivery. The IQM system's ability to monitor leaf gap constancy throughout a dynamic beam delivery and its sensitivity to variations in leaf gap were evaluated. Similar to the previous section, the MLC bank was divided into four sections. A two‐segment dynamic field was developed to deliver 112 MU while sliding a 1 × 10 cm^2^ MLC defined slit, back and forth, between ‐7 cm to +7 cm leaf positions at maximum leaf velocity of 2.5 cm/s. The X jaws were positioned at ± 7.5 cm, while the Y diaphragms were confined to the length of the narrow slit. The field delivery was repeated for other leaf sections at gantry zero and a baseline was established. Measurements were repeated at other cardinal gantry angles and compared to baseline to assess the effect of gravity on dynamic beam delivery. The IQM's sensitivity to leaf gap variation was tested by changing the dynamic slit width by −3 mm to +3 mm in 1 mm increment and comparing these measurements to the baseline signal.

## 3 RESULTS

### 3.1 Photon beam output

The sensitivity of the IQM system to typical daily Linac output variation was assessed for all beam energies using a 20 × 20 cm^2^ field size. The simulated output variation measurements for the IQM and the reference ion chamber were performed by changing the set MU with respect to 100 MU, as summarized in Table 1. Both detectors were able to measure the ± 0.5% and ± 1.0% photon beam output variation to within 0.1% accuracy across all photon beam energies. The percent standard deviation for all measurements were within 0.1% for both detectors. From the results below, it can be concluded that the IQM system is sensitive to the small day‐to‐day variations of the beam output and therefore can be used reliably for routine Linac output constancy measurements.

**TABLE 1:** Beam output variation detection by the Integral Quality Monitor (IQM) system.

	6 MV		10 MV		18 MV		6 FFF		10 FFF	
MU	IQM	REF	IQM	REF	IQM	REF	IQM	REF	IQM	REF
−1.0%	−0.90	−1.00	−0.97	−1.03	−0.95	−0.98	−1.04	−1.05	−0.96	−1.01
−0.5%	−0.42	−0.48	−0.49	−0.51	−0.45	−0.47	−0.54	−0.53	−0.49	−0.50
+0.5%	+0.55	+0.51	+0.51	+0.49	+0.53	+0.51	+0.45	+0.47	+0.58	+0.58
+1.0%	+1.07	+1.02	+1.01	+0.98	+1.02	+1.04	+0.96	+0.93	+1.01	+1.03

### 3.2 Photon beam output versus gantry angle

The beam output for all beam energies were measured simultaneously for a 20 × 20 cm^2^ field size by the IQM system and reference ion chamber at cardinal gantry angles, and were compared to their respective readings at gantry zero. The percent difference of readings with respect to the reading at gantry zero for each detector and beam energy are presented in Table 2. Signals measured by both detectors were reproducible to within a standard deviation of 0.1%. The reference ion chamber measured a maximum of 0.59% beam output variation at gantry angle 180° for all beam energies. This may be mainly due to the partial beam attenuation caused by the plastic frame on which the cylindrical phantom was resting. In contrast, for increasing beam energies, IQM detected larger variations of output as a function of gantry angles, with a maximum difference of 1.96% for 18 MV beam at gantry angle 180°.

**TABLE 2:** Linac output variation with gantry angle for various beam energies.

	6 MV		10 MV		18 MV		6 FFF		10 FFF	
Gantry	IQM	REF	IQM	REF	IQM	REF	IQM	REF	IQM	REF
180°	−0.24	−0.59	−1.07	−0.29	−1.96	−0.28	−0.08	−0.30	−0.78	−0.23
90°	−0.33	−0.27	−0.84	−0.14	−1.28	−0.08	−0.24	−0.06	−0.51	−0.04
−90°	−0.08	−0.22	−0.64	0.06	−1.19	0.00	−0.08	0.13	−0.45	0.06

Abbreviation: IQM, Integral Quality Monitor.

The cause of the apparent output variation as a function of gantry angle measured with the IQM system for higher energy beams is not entirely clear. However, considering the inadequate buildup depth of the IQM chamber entrance electrode plate, which is 1 mm of Aluminum, for higher energy beams, it can be speculated that a small change in mean contaminating electron energy at various gantry angles (due to reasons to be investigated) could provide significantly different chamber response. Consequently, a buildup depth approximately equal to the *d*_max_ depth could possibly reduce this effect. With this hypothesis, the above experiment was repeated, adding 3 mm of copper sheets on the entrance surface of the IQM chamber and taking repeated measurements. The results of the modified measurements are shown in Table 3. As shown, the variations of output as a function of gantry angles have reduced significantly and are in close agreement to those measured by the reference cylindrical chamber. While, for routine constancy measurements with the IQM system it will not be necessary to add the copper plates, this experiment provides an initial explanation of the apparent variations of the output in the IQM measurement.

**TABLE 3:** Linac output variation with gantry angle for various beam energies with 3 mm added aluminum plate.

	6 MV		10 MV		18 MV		6 FFF		10 FFF	
Gantry	IQM	REF	IQM	REF	IQM	REF	IQM	REF	IQM	REF
180°	0.33	−0.29	0.18	−0.18	0.08	−0.35	0.21	−0.17	0.26	−0.22
90°	0.09	−0.07	0.23	−0.07	−0.08	−0.11	0.20	−0.02	0.07	0.07
−90°	0.00	0.04	0.02	0.02	0.06	−0.11	0.04	0.07	0.12	0.15

Abbreviation: IQM, Integral Quality Monitor.

### 3.3 Photon beam output versus dose‐rate

**FIGURE 4:**
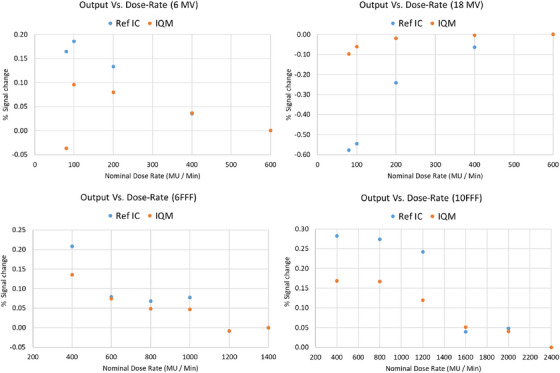
Linac output variation with nominal dose‐rate (normalized at maximum dose rate) for various beam energies measured by Integral Quality Monitor (IQM) and reference chamber (Ref IC). The percent change in machine output for a 10 × 10 cm^2^ beam measured by both detectors agreed to within 0.12%. The pattern in output variation was unique to each beam energy which was captured by IQM as well as the reference chamber.

Linac output variation with dose‐rate was measured by both IQM system and ref ion chamber as shown in Figure 4. Both IQM and the reference chamber were able to detect slight output variation with dose rate for each beam energy. The pattern in inherent Linac output variation measured by reference chamber was different for each beam energy. These output variation patterns were also captured by the IQM system to within 0.12% agreement. Results suggest that IQM's system sensitivity to output variation with dose‐rate is similar to the reference ion chamber, indicating its suitability for output constancy measurement as a function of dose rate.

### 3.4 Beam symmetry

**FIGURE 5:**
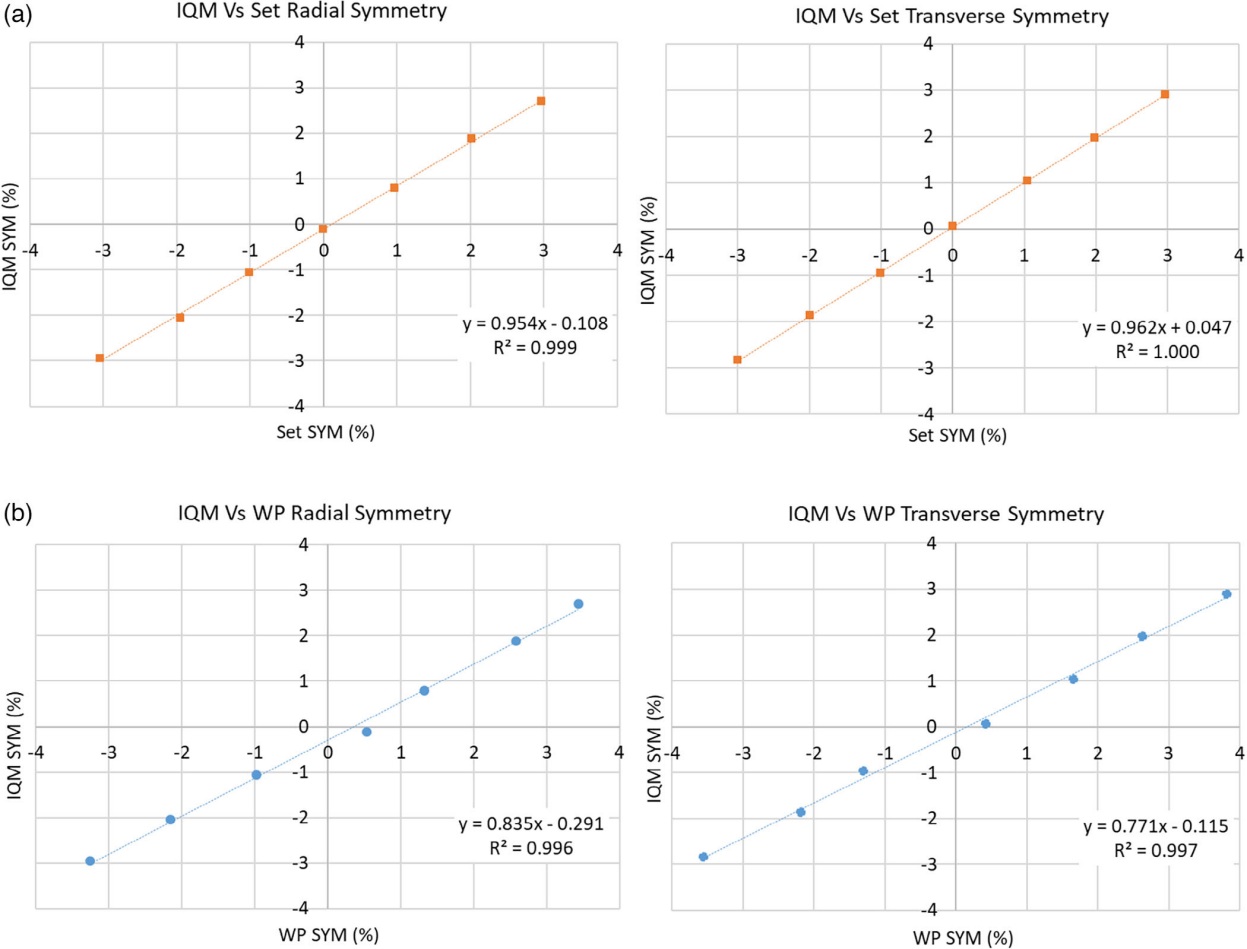
IQM chamber's sensitivity to beam symmetry variation for a 10 MV beam. (a) A linear relationship with minimal offset was observed when radial and transverse symmetry measured by IQM system $\left(IQM_{SYM}\right)$ was compared to those measured by Linac monitoring chamber $\left(Set_{SYM}\right)$. IQM system and Linac monitoring chamber have similar sensitivity to beam symmetry changes. (b) $IQM_{SYM}$ compared to beam symmetry measured by water phantom scanner $\left(WP_{SYM}\right)$ for field 35 × 35 cm^2^ at depth *d*_max_. IQM system was less sensitive (< 0.25%) to beam symmetry variation when compared to WP due to signal averaging effect of 4 × 4 cm^2^ field size. The small offset in $WP_{SYM}$ may be due to WP setup error. IQM, Integral Quality Monitor.

The sensitivity of the IQM system to beam symmetry variations was explored by deliberately changing the beam symmetry of a 10 MV beam. The beam symmetry measured by IQM ($IQM_{SYM})$ was compared to those reported by the Linac beam monitoring chamber ($Set_{SYM})$, and water phantom scans $\left(WP_{SYM}\right)$ for a 30 × 30 cm^2^ field size shown by Figure 5.

The results suggest the IQM system has a highly linear response to beam symmetry variation reported by the Linac monitoring chamber in both radial and transverse axes (*R* ^2^ = 0.999 and 1.000). The linear equations can translate beam asymmetry measured by the IQM system to that measured by the Linac beam monitoring ion chamber. As shown, the IQM system measured a beam asymmetry of 0.954 ± 0.01% and 0.962 ± 0.04% change for every 1% change detected by the monitoring ion chamber in radial and transverse axes, respectively. This near one‐to‐one relationship is due to similarities in beam symmetry measurement by both detectors. The Linac monitoring chamber uses the dose information measured by different sectors within the chamber, whereas the IQM transmission chamber simulates those sectors through rotation of an off‐axis 4 × 4 cm^2^ opening. As a result, both detectors are sampling an area of the beam profile. The radial and transverse *Y*‐intercepts were −0.108 ± 0.02% and 0.047 ± 0.01% respectively. The minimal offset in the fitted line is perhaps a result of the IQM system's slight misalignment with the Linac monitoring chamber and beam center.

Similarly, a linear relationship was found between $IQM_{SYM}$ and $WP_{SYM}$, and the equations can be used to translate beam asymmetry measured by the IQM system to those measured using WP. The IQM system detected a change of 0.835 ± 0.023%, and 0.771 ± 0.019% for every 1% change detected by WP scans in the radial and transverse axes respectively. These sensitivity factors can be used to set appropriate tolerances around IQM beam symmetry measurements. For example, a recommended tolerance of ± 2% for WP measurements translates to approximately ± 1.6% tolerance for IQM beam symmetry measurements. The IQM system is less sensitive to beam symmetry variation by approximately 0.2% when compared to WP. This reduction in sensitivity is explained by IQM's large area averaging effect when compared to CC13 ion chambers, which is a closer representation of point dose measurement.

The radial and transverse *Y*‐intercepts were −0.291 ± 0.054% and −0.115 ± 0.047%, respectively. This relatively larger offset between $IQM_{SYM}$ and $WP_{SYM}$ measurements can be caused by an imperfect WP setup, as a slight tilt or misalignment in scanning arms can result in small errors in beam symmetry measurement.

### 3.5 Beam flatness

**FIGURE 6:**
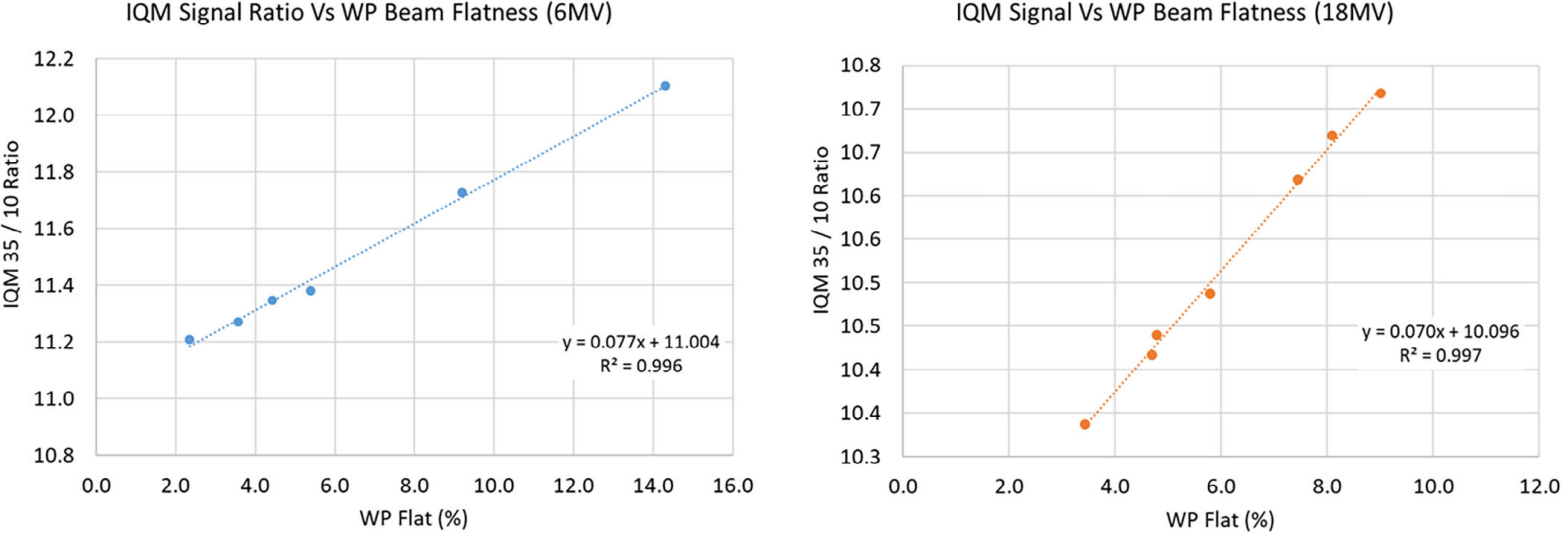
IQM sensitivity to beam flatness variation for 6 MV and 18 MV beams. Beam flatness measured by the IQM system (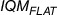) changed linearly with beam flatness measured in water phantom (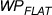) for a 35 × 35 cm^2^ at *d*_max_ when beam energy was varied. IQM, Integral Quality Monitor.

The sensitivity of the IQM system to beam flatness variation was explored for 6 and 18 MV beams using the two‐ field technique and compared to beam flatness measured in water phantom. Figure 6 demonstrates the relationship between change in 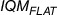 and 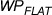 with respect to their baseline values for 6 and 18 MV beam. The data suggests 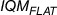 changes linearly with 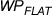 (*R* ^2^ = 0.996 for 6 MV data and *R* ^2^ = 0.997 for 18 MV data). IQM signal ratio changed by 0.690% and 0.684% for every 1% change in beam flatness measured by WP for 6 and 18 MV beam respectively. Similar to the section above, these measured IQM sensitivity specifications can be used to set tolerances on IQM beam flatness constancy measurements that satisfy the protocol followed for the clinical center.

### 3.6 Static MLC positioning accuracy

IQM's sensitivity to random and systematic leaf positioning error was investigated using a picket fence test with 1 × 10 cm^2^ slits. Results show IQM signal changed by 5.79 ± 0.23% for 1 mm systematic leaf bank error. A 3 mm random leaf positioning error introduced a 1.88 ± 0.13% change in IQM signal for the outer 10 mm wide MLCs on average, and 0.95 ± 0.12% change for the inner 5 mm wide MLCs. While the IQM system is suitable to detect a leaf bank error of 1 mm, the results imply that the system lacks the sensitivity to detect a 1 mm random MLC positioning error.

### 3.7 Dynamic MLC positioning accuracy

**FIGURE 7:**
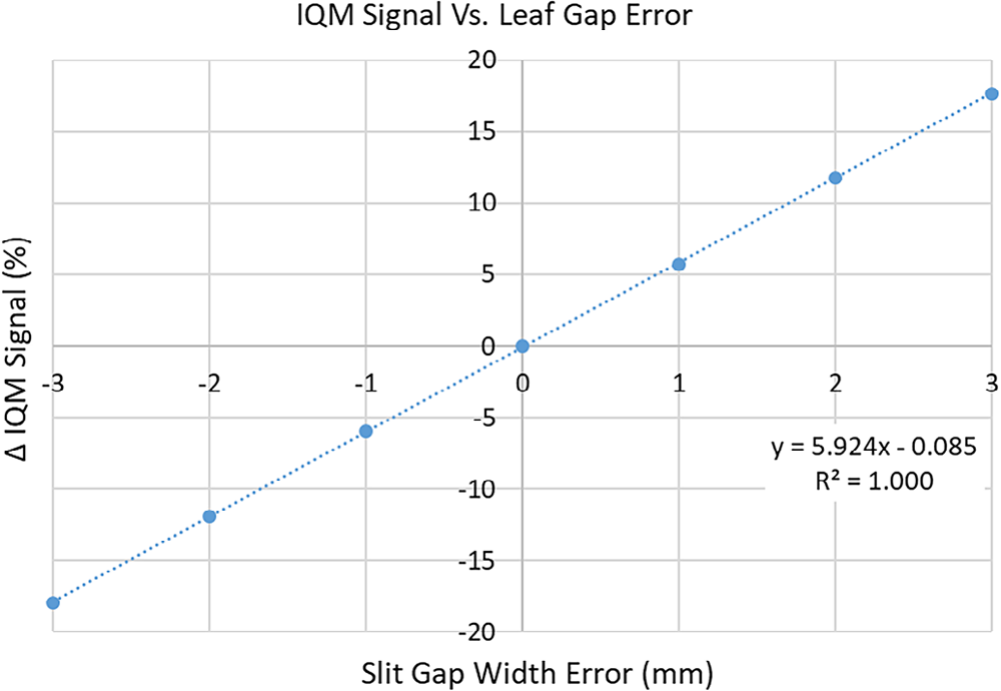
Change in IQM signal during a dynamic MLC beam delivery with varying gap. The MLC gap for a 1 × 10 cm^2^ was opened and closed by up to ±3 mm in 1 mm increment. IQM signal changed by 5.924% for every 1 mm change in narrow slit gap. IQM, Integral Quality Monitor; MLC, multileaf collimator.

The IQM system's sensitivity to dynamic MLC positioning error was studied using a 1 × 10 cm^2^ sliding window slit. Figure 7 shows a plot of the change in IQM signal for gap width errors ranging from ‐3 mm to 3 mm. The IQM response to change in dynamic gap was highly linear with a sensitivity of 5.92 ± 0.02% per 1 mm change in leaf gap.

The signals acquired at each cardinal angle were compared to those at gantry 0° shown in Table 4. The relative change in IQM signal was < 1% for all four leaf sets and gantry angles.

**TABLE 4:** IQM dynamic MLC signal deviation at cardinal gantry angles with respect to gantry zero.

*% Difference in IQM signal with respect to Gantry Zero*				
Row #	Leaf pairs	Gantry 180°	Gantry 90°	Gantry 270°
1	51–60	0.45 ± 2.40E‐03%	0.13 ± 3.25E‐04%	−0.08 ± 1.51E‐03%
2	31–50	0.61 ± 2.84E‐04%	0.28 ± 7.53E‐04%	−0.42 ± 9.28E‐04%
3	11–30	0.55 ± 1.72E‐04%	0.31 ± 1.73E‐04%	−0.29 ± 8.44E‐04%
4	1–10	0.90 ± 2.02E‐04%	0.75 ± 2.33E‐04%	−0.22 ± 5.10E‐04%

Abbreviations: IQM, Integral Quality Monitor; MLC, multileaf collimator.

IQM signals measured at gantry 90° were greater than those at gantry zero, and the signals acquired at gantry 270° were smaller than gantry 0°. This may indicate a slight increase in gap width at gantry 90°, and decrease in leaf gap at gantry 270°, due to gravitational effects on one of the leaf banks.

The largest variations in the IQM signal were observed at gantry 180°, where leaves are not under influence of gravity. These variations can be due to small sub‐millimeter shift in Y Jaws. The Y jaws of a Varian TrueBeam Linac are divergence matched, are situated on a ramp, and move along a circular trajectory. Y Jaw positioning can be affected at gantry angles 0° and 180°, where they move with or against gravity.

## 4 DISCUSSION

The feasibility of using the IQM system for dosimetric QA of Linacs has been investigated in this multicenter study. The dosimetric QA tests selected cover most of the routine QA tests recommended by radiotherapy communities of practice. Due to the simplicity and robustness of equipment setup, as well as the potential of automation of data acquisition and analysis, the tests can be performed more easily and frequently as may be desired for the modern practice of dynamic radiotherapy.

The sensitivity of the IQM system to typical daily variation of beam output has been shown to be similar to that of the Farmer type ion chamber, making the IQM system suitable for beam output constancy measurements. Temperature and pressure corrected IQM signal for a large field (e.g., 20 × 20 cm^2^ or larger) can be scaled to provide a beam output constancy in cGy/MU, as shown by Equation (5) below

(5) $$D\left(\frac{cGy}{MU}\right)=K\times IQM_{20\times 20}$$

where *K* is a proportionality constant, which can be determined following an absolute dose output measurement consequent to the IQM signal measurement.

As previously stated, IQM system response to dose was found to be highly linear. This may provide an opportunity to assess the Linac beam delivery control system by evaluating photon output linearity. Signals can be acquired for various beam MU with the IQM system to evaluate variations in counts‐per‐MU as a function of total MU to assess Linac beam control system functionality. It has been recommended by AAPM TG‐142^3^ to check dose linearity for segmented step‐and‐shoot fields over the clinical range of MU‐per‐segment, as well as the linearity for static fields on annual basis, and that the ratio of dose measured using static field and those measured using segmented field must be within 2%. In the interest of increasing beam delivery precision, more frequent dose linearity measurements, for example monthly, may prove to be beneficial, especially for low MU fields or segments. The IQM system can streamline this process since it has the ability to report the signal measured for each beam segment. An approach we propose using the IQM system is to measure a set of reference signals at various total beam MU using static field on annual basis. The same set of static fields can then be transformed to a segmented step‐and‐shoot field and delivered on monthly basis. The ratio of dose measured using static and segmented field must be within the recommended tolerances.

The beam output of a Linac can slightly vary with the variation of nominal dose rates for a given beam energy. It is also observed that the patterns in beam output variation with dose rate, for a given beam energy, differ from one Linac to another Linac of the same type and model. This is likely due to inherent characteristic of the Linac's dose monitoring system and its control of beam pulses. However, in this investigation, both the IQM system and the reference ion chamber were able to detect the same pattern in output variation with nominal dose rate for all beam energies, implying that the IQM system can effectively monitor the constancy of output as a function of dose rate. Further explanation of such behavior can be complex, and is considered to be out of the scope of this paper.

The reason for the apparent variations in the beam output observed by IQM system as a function of gantry angles for higher beam energies can be attributed to the inadequate equivalent build‐up depth of the entrance electrode plate. However, the IQM system could still be used to measure output constancy as a function of gantry angle. Any significant deviation of constancy would flag any potential issues with changes in beam spectrum or other dosimetric or mechanical issues. Another potential method of measuring the output variation as a function of gantry angle, using the IQM system, is to deliver a uniform open field arc, and plotting the signal rate (count‐per‐second) as function of the gantry angle reported by IQM's onboard inclinometer. The curve can then be compared to a reference curve that is acquired annually.

Although the IQM system utilizes a single transmission chamber and provides one signal per beam segment, it was shown that the beam symmetry constancy measurement is still possible by using small off‐axis beam segments at various collimator rotations. IQM sensitivity to beam symmetry error was found to be slightly lower than that of the WP scans, however the linear relationship can be used to set a tolerance around IQM symmetry measurements that is consistent with those defined by various protocols. Although a 10 MV beam was used for the beam symmetry experiment in this report, the technique can be applied for other beam energies.

Beam flatness was measured using a two‐field ratio technique, and the models from Figure 6 can be used to set tolerances when performing a beam energy check. A conventional approach of measuring the beam flatness by sampling the beam central region and an off‐axis region using separate beam apertures has not been considered in this study because of the uncertainties in the jaw positioning. The jaws (secondary collimators) of Varian TrueBeam Linacs have a positional uncertainty of 1 mm, and hence can introduce significant variations in the “dose‐area product” signal of IQM and can consequently produce a significant change in flatness values.

Although, it is feasible to determine the beam symmetry and flatness by the IQM system effectively, it is recognized that the procedure requires multiple field segment measurements, as opposed to a simple measurement with a real‐time beam adjustment opportunity provided by a 2‐D array detector system. The IQM system, however, is much simpler in design, and does not require calibration, as needed for a 2‐D array device. Furthermore, the ability to perfor beam output measurements without the need of an external electrometer, and the ability of MLC positioning constancy measurement, makes the IQM system an effective alternative QC tool for a Linac.

In this work with the IQM system, we investigated the MLC leaf positioning test with a dosimetric equivalent of the Picket‐Fence test, and similarly assessed the dynamic beam delivery test with a sliding window of a slit aperture. The IQM system was found to be relatively insensitive to a 1 mm random leaf errors, but, it can detect a 1 mm bank error for both static and dynamic beam delivery. Theoretically, a 1 mm systematic MLC bank error for a 1 × 10 cm^2^ slit should change the aperture area, and consequently the IQM signal by ± 10%. Since the IQM system integrates the signal generated by the entire chamber area, including the beam transmission through the Jaws and MLC leaves, the sensitivity for a 1 mm bank error is reduced from the theoretical value of 10% to nearly 5.8%.

Each Linac constancy test with the IQM system was studied individually to gain a deep understanding of IQM system's performance and its characteristics for each Linac test. A protocol can be developed by creating a series of static and IMRT test fields that can be delivered on daily, weekly, and monthly basis. The frequency of the tests is to be decided by the clinical institute based on their clinical practice.

## 5 CONCLUSION

This work investigated the feasibility of using the transmission ion chamber of the IQM system for routine dosimetric QA tests of Linacs. The results suggest that the sensitivity of the IQM system in detecting variations of the routine test parameters including the beam output, symmetry, flatness, and MLC bank positioning tests is similar to those of the conventional equipment, and therefore, the system can be utilized as a routine QA tool. Due to the automatic mode of operation of the IQM system, a selected set of test fields can be incorporated in a dynamic MLC sequence to acquire and analyze the measurement data in an efficient way, enabling the user in the clinic to run these tests routinely, and if required, with increased frequency.

Future work will involve development of a software module dedicated to Linac QA to analyze IQM results, and an efficient reporting system.

## AUTHOR CONTRIBUTIONS

This research project was conceived by Mohammad Islam (MI), and Luis Fong Santos (LFS). The list of experiments for the project was planned by all the authors. The initial tests were designed, run, and optimized by Makan Farrokhkish (MF). Subsequently, some of these tests were run by Andrew Veres (AV) and John DeMarco (JDM) at their respective institute for reproducibility and consistency. MF has refined the design of the experiments and analyzed the results with input from all the authors. The manuscript is written by MF, reviewed by all the authors; revised to its present form by MF and MI.

## CONFLICT OF INTEREST STATEMENT

Several authors of this paper are in a royalty relationship with manufacturer of the IQM system, iRT.
